# P10-11 The effect of a pedometer-based intervention across two years, in people with type 2 diabetes and prediabetes - a compositional data analysis

**DOI:** 10.1093/eurpub/ckac095.150

**Published:** 2022-08-29

**Authors:** Kristina Larsson, Philip Von Rosen, Jenny Rossen, Unn-Britt Johansson, Maria Hagströmer

**Affiliations:** 1 Sophiahemmet University, Stockholm, Sweden; 2 Karolinska Institutet, Stockholm, Sweden

**Keywords:** Accelerometer, CoDA, Diabetes, Randomized controlled trial, Longitudinal data

## Abstract

**Background:**

For people with prediabetes and type 2 diabetes it is important to be regularly physical active. Increasing and maintaining physical activity (PA) can be challenging. The aim of this study was to evaluate the effects of a pedometer-based intervention on PA, with a compositional data analysis (CoDA) approach, in individuals with prediabetes or type 2 diabetes.

**Methods:**

Longitudinal data on 188 participants with prediabetes and type 2 diabetes (40% female, mean age = 64.1 years) from a three-armed randomized controlled trail, the Sophia Step Study, was used. The three groups were a multi?component group (self?monitoring of steps with a pedometer, together with group (12 occasions) and individual counselling (10 occasions)), a single?component group (self?monitoring of steps with a pedometer, without counselling) and a standard care group. PA (moderate-to-vigorous PA (MVPA), light-intensity PA (LIPA) and sedentary behaviour (SB)) during the awake time were measured with ActiGraph GT1M accelerometer at baseline, 6, 12, 18 and 24 months. Relative time in MVPA, LIPA and SB for each participant at each measurement point was calculated by using the CoDA approach. Linear mixed models were used to evaluate the intervention effect between the three groups on the relative time in MVPA, LIPA and SB over the two-year period.

**Results:**

In total, 41% had ≤30 min MVPA at baseline. Significant group by time interactions were found for the multi-component group and the standard care group for the relative time in MVPA, at 6, 18 and 24 months. Differences in predicted group means between the groups were 1.2% at 6 months, 1.3% at 18 months and 0.9% at 24 months. Significant group by time interactions were also found for the single-component group and the standard care group for the relative time in MVPA at 24 months, with a difference in predicted group means of 0.8%. No significant interactions for LIPA or SB were found.

**Conclusions:**

The multi-component group and the single-component component group maintained their relative time in MVPA, while the standard care group decreased their relative time in MVPA over the two-year period. This indicate that the Sophia Step Study intervention can prevent a decrease in MVPA in people with prediabetes and type 2 diabetes.

